# The mitochondrial antioxidant SS-31 increases SIRT1 levels and ameliorates inflammation, oxidative stress and leukocyte-endothelium interactions in type 2 diabetes

**DOI:** 10.1038/s41598-018-34251-8

**Published:** 2018-10-26

**Authors:** Irene Escribano-Lopez, Noelia Diaz-Morales, Francesca Iannantuoni, Sandra Lopez-Domenech, Aranzazu M de Marañon, Zaida Abad-Jimenez, Celia Bañuls, Susana Rovira-Llopis, Jose R Herance, Milagros Rocha, Victor M Victor

**Affiliations:** 1 Service of Endocrinology, University Hospital Doctor Peset, Foundation for the Promotion of Health and Biomedical Research in the Valencian Region (FISABIO), Valencia, Spain; 2 Medical Molecular Imaging Research Group, Vall d’Hebron Research Institute (VHIR), CIBBIM Nanomedicine, Passeig de la Vall d’Hebron, Barcelona, Spain; 3 CIBERehd - Department of Pharmacology, University of Valencia, Valencia, Spain; 4 Department of Physiology, University of Valencia, Valencia, Spain

## Abstract

There is growing focus on mitochondrial impairment and cardiovascular diseases (CVD) in type 2 diabetes (T2D), and the development of novel therapeutic strategies in this context. It is unknown whether mitochondrial-targeting antioxidants such as SS-31 protect sufficiently against oxidative damage in diabetes. We aimed to evaluate if SS-31 modulates SIRT1 levels and ameliorates leukocyte-endothelium interactions, oxidative stress and inflammation in T2D patients. Anthropometric and metabolic parameters were studied in 51 T2D patients and 57 controls. Production of mitochondrial reactive oxygen species (ROS), mitochondrial membrane potential, glutathione content, leukocyte-endothelium interactions, NFκB-p65, TNFα and SIRT1 levels was measured in leukocytes treated or not with SS-31. We observed increased mitochondrial ROS production that was restored by SS-31 treatment. SS-31 also increased mitochondrial membrane potential, glutathione content, SIRT1 levels and leukocyte rolling velocity and reduced rolling flux and adhesion in T2D patients. NFκB-p65 and TNFα, which were enhanced in diabetic patients, were also reduced by SS-31 treatment. Our results reveal that SS-31 exerts beneficial effects on the leukocytes of T2D patients by reducing oxidative stress, leukocyte-endothelium interactions, NFκB and TNFα and by increasing SIRT1 levels. These actions support its use as a potential agent against CVD risk.

## Introduction

Type 2 diabetes (T2D) is an increasingly prevalent disease and a serious health problem worldwide, as it can markedly reduce life expectancy^1,2^. T2D is associated with diverse cardiovascular risk factors, such as insulin resistance, obesity, hypertension, dyslipidaemia and non-alcoholic fatty liver disease, as well as platelet and homeostatic abnormalities that increase the risk of thrombosis^3^. As a consequence, T2D is implicated in a series of disorders, particularly cardiovascular diseases (CVD), though the underlying mechanisms are yet to be determined.

Type 2 diabetes has been associated with enhanced production of reactive oxygen species (ROS) and, consequently, an alteration of redox state and cellular homeostasis. Mitochondria are key organelles in the regulation of the metabolism, the major site of ATP production, and one of the main sources of ROS. In this sense, class III histone deacetylase sirtuin-1 (SIRT1) is a key protein which controls pathways that regulate the metabolic components of mitochondria^4^. Furthermore, SIRT1 directly interacts with and deacetylates the peroxisome proliferator–activated receptor γ coactivator-1α (PGC-1α)^5,6^, the master regulator of mitochondrial activity and a main player in mitochondrial biogenesis and function.

Mitochondria are particularly vulnerable to hyperglycaemic conditions, enhancing ROS production and oxidative stress^7,8^. In this regard, mitochondrial dysfunction and oxidative stress have been related to the onset of T2D and insulin resistance^9^. Indeed, our group has demonstrated impaired mitochondrial function and subsequent enhancement of ROS production in diabetic patients, as well as changes in mitochondrial membrane potential and a reduction of antioxidant content^10^.

Inflammation plays a vital role in host defences, since immune cells release pro-inflammatory cytokines, such as tumour necrosis factor alpha (TNFα), to protect against injury; for example, several studies suggest that inflammation is a key player in the pathogenesis of some glucose disorders^11^. During the progression of T2D, a chronic and low-grade inflammatory response takes place due, in part, to the effects of hyperglycaemia on white blood cells^12,13^. It has been demonstrated that the nuclear factor kappa B (NFκB), a central regulator of immunity, inflammation and cell survival, is activated under these conditions^14–16^. This inflammatory state involves an enhanced adhesion of leukocytes to the surface of the endothelium, after which they migrate in order to destroy pathogens by generating production of ROS. Given that enhanced ROS production under oxidative stress contributes to the mitochondrial injury that promotes endothelial dysfunction and, in turn, leukocyte adhesion, inflammation, thrombosis and smooth muscle cell proliferation^17^, the search for novel therapies that ameliorate mitochondrial oxidative stress in metabolic diseases such as T2D is paramount.

SS-31 (D-Arg-2′6′-dimethylTyr-Lys-Phe-NH_2_) is a cell-permeable mitochondria-targeted antioxidant tetrapeptide with an alternating aromatic-cationic structure. SS-31 can scavenge mitochondrial ROS, thereby promoting mitochondrial function and inhibiting mitochondrial permeability transition^18,19^. These effects are due to the dimethyltyrosine contained within SS-31, and are not exerted by other related peptides such as SS-20, which lack this structure^20^. As a small peptide, SS-31 is water-soluble and offers other advantages, such as the capacity to target and concentrate at the inner mitochondrial membrane in a membrane potential-independent manner and to protect against mitochondrial depolarization^21^.

In the present study, we investigate the potential therapeutic benefits of SS-31 with respect to SIRT1 levels, oxidative stress parameters and leukocyte-endothelial interactions and evaluate its impact on NFκB in leukocytes from T2D patients.

## Results

### Anthropometric and metabolic parameters

We evaluated 51 T2D patients and compared them with 57 healthy subjects (Table 1). Non-statistical differences were observed among the groups with respect to sex, age, and diastolic blood pressure. However, compared to the control group, diabetic patients displayed higher weight (p < 0.01), body-mass index (BMI), waist circumference, systolic blood pressure (BP), HOMA-IR, insulin, HbA1c and fasting glucose levels (p < 0.001). T2D patients showed a typical lipid profile of reduced levels of HDL-c (p < 0.001) and elevated levels of triglycerides (p < 0.01) with respect to controls. However, levels of low-density lipoprotein cholesterol (LDL-c) and total cholesterol were lower T2D patients than in controls (p < 0.001) due lipid-lowering medication (67% were taking statins, 12% fibrates and 2.4% ezetimibe). In terms of the oral antidiabetic treatment received by patients, 61.4% were treated with metformin, 33.3% with DPP-4 inhibitors, 15.9% with GLP-1 agonists, 6.8% with SGLT2 inhibitors, 4.5% with sulphonylureas and 2.3% with glitazones. In comparison with control subjects T2D patients displayed a more pro-inflammatory state, manifested by their levels of high-sensitive C-reactive protein (hs-CRP) (p < 0.01). Statistical significance remained after adjustment for BMI, with the exception of triglycerides and hs-CRP (p < 0.01).

**Table 1:** Baseline characteristics of the subjects.

Parameter (Unit) [Reference Interval]*	Control	Type 2 Diabetes	p Value	BMI adjusted p Value
N	57	51	—	—
Male %	42.1	51.9	ns	—
Age (years)	53.7 ± 7.7	57.1 ± 10.3	ns	—
Weight (kg)	72.8 ± 18.0	84.7 ± 16.4	<0.01	—
BMI (kg/m^2^)	25.7 ± 4.1	30.6 ± 5.5	<0.001	—
Waist (cm)	87.7 ± 12.7	103.5 ± 11.7	<0.001	—
Systolic BP (mmHg)	124 ± 20	146 ± 14	<0.001	<0.001
Diastolic BP (mmHg)	75 ± 10	67 ± 31	ns	ns
Fasting Glucose (mg/dl) [70–105]	94.1 ± 9.4	153.7 ± 45.0	<0.001	<0.001
Insulin (µUI/mL) [2.0–14.0]	7.19 ± 2.62	12.56 ± 6.57	<0.001	<0.05
HOMA-IR [0.0–3.8]	1.63 ± 0.73	4.55 ± 2.73	<0.001	<0.001
HbA1c (%) [4.0–5.6]	5.3 ± 0.3	7.1 ± 1.2	<0.001	<0.001
Total cholesterol (mg/dl) [80–200]	203.7 ± 34.5	158.7 ± 39.1	<0.001	<0.001
HDL-c (mg/dl) [35–70]	57.2 ± 19.7	43.5 ± 9.8	<0.001	<0.01
LDL-c (mg/dl) [40–150]	126.5 ± 29.5	87.0 ± 31.7	<0.001	<0.001
Triglycerides (mg/dl) [30–150]	90.5 (62.8; 150.5)	132.0 (92.8; 164.8)	<0.01	ns
hs-CRP (mg/l) [0.0–1.69]	1.17 (0.44; 2.17)	2.45 (1.22; 5.39)	<0.01	ns

Data are shown as mean ± SD for parametric variables and were compared by a Student’s t test, or as median (25^th^ and 75^th^ percentiles) for non-parametric variables and were compared by a Mann–Whitney U test. A univariate general linear model was used to adjust changes by BMI. To compare proportions among groups, Chi-Square test was used.

*Reference intervals according to the service of clinical analysis of our hospital were taken into account for the interpretation of clinical laboratory results.

### Mitochondrial Function and Oxidative Stress

**Figure 1:**
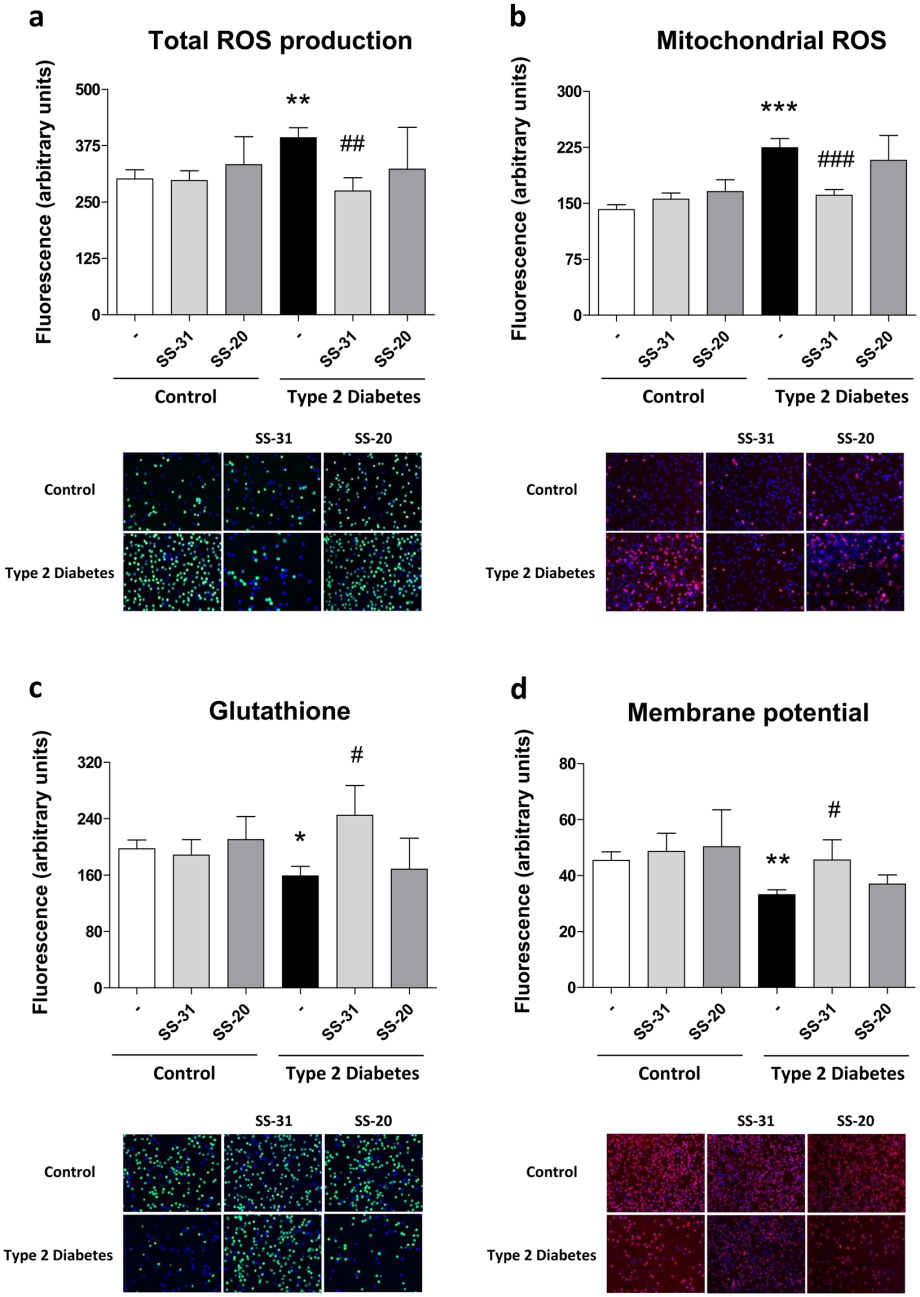
Evaluation of oxidative stress parameters in leukocytes from T2D patients and controls in the absence and presence of SS-31 (30 min, 100 nM) (**a**) ROS production measured as DCFH-DA fluorescence. (**b**) Mitochondrial ROS production measured as MitoSOX fluorescence. (**c**) GSH levels measured as CMFDA fluorescence. (**d**) Mitochondrial membrane potential measured as TMRM fluorescence. *p < 0.05 **p < 0.01 ***p < 0.001 with regard to control group ^#^p < 0.05 ^##^p < 0.01 ^###^p < 0.001 *vs*. non-treated T2D group. All these results are shown together with representative fluorescence microscopy images.

Higher levels of total ROS production were observed in the leukocytes of T2D patients (p < 0.01, Fig. 1a). In addition, diabetic patients exhibited higher levels of mitochondrial ROS production than control subjects (p < 0.001, Fig. 1b). Interestingly, treatment with the mitochondria-targeted antioxidant SS-31 reduced both parameters in the leukocytes of T2D patients (p < 0.01 and p < 0.001, respectively). Confocal microscopy in human leukocytes revealed MitoSOX (red fluorescence) localization in the mitochondria. Furthermore, rotenone (a complex I inhibitor) induced an increase in Mitosox fluorescence indicating mitochondrial superoxide production (see Supplementary Fig. S1).

CMFDA fluorescence, which is proportional to the content of the free thiol form of GSH in leukocytes, was lower in T2D patients than in healthy subjects (p < 0.05, Fig. 1c). SS-31-treated leukocytes of T2D patients exhibited significantly higher levels of the antioxidant GSH (p < 0.05) than non-treated leukocytes. Furthermore, TMRM fluorescence, which is employed to estimate mitochondrial membrane potential, was significantly lower in diabetic patients than control subjects (p < 0.01, Fig. 1d). Treatment with the mitochondria-targeting antioxidant SS-31 increased mitochondrial membrane potential in diabetic patients (p < 0.05) with respect to basal conditions. In the supplementary data, confocal microscope images are appended to show that TMRM fluorescence corresponds with a mitochondria pattern (see Supplementary Fig. S1), showing a decrease in the fluorescence intensity consequently to mitochondrial depolarization with CCCP addition.

SS-31 did not have a notable influence on leukocytes of control subjects and SS-20 did not modify oxidative stress parameters (Fig. 1a–d).

### Evaluation of SIRT1 protein levels and sirt1 gene expression

**Figure 2:**
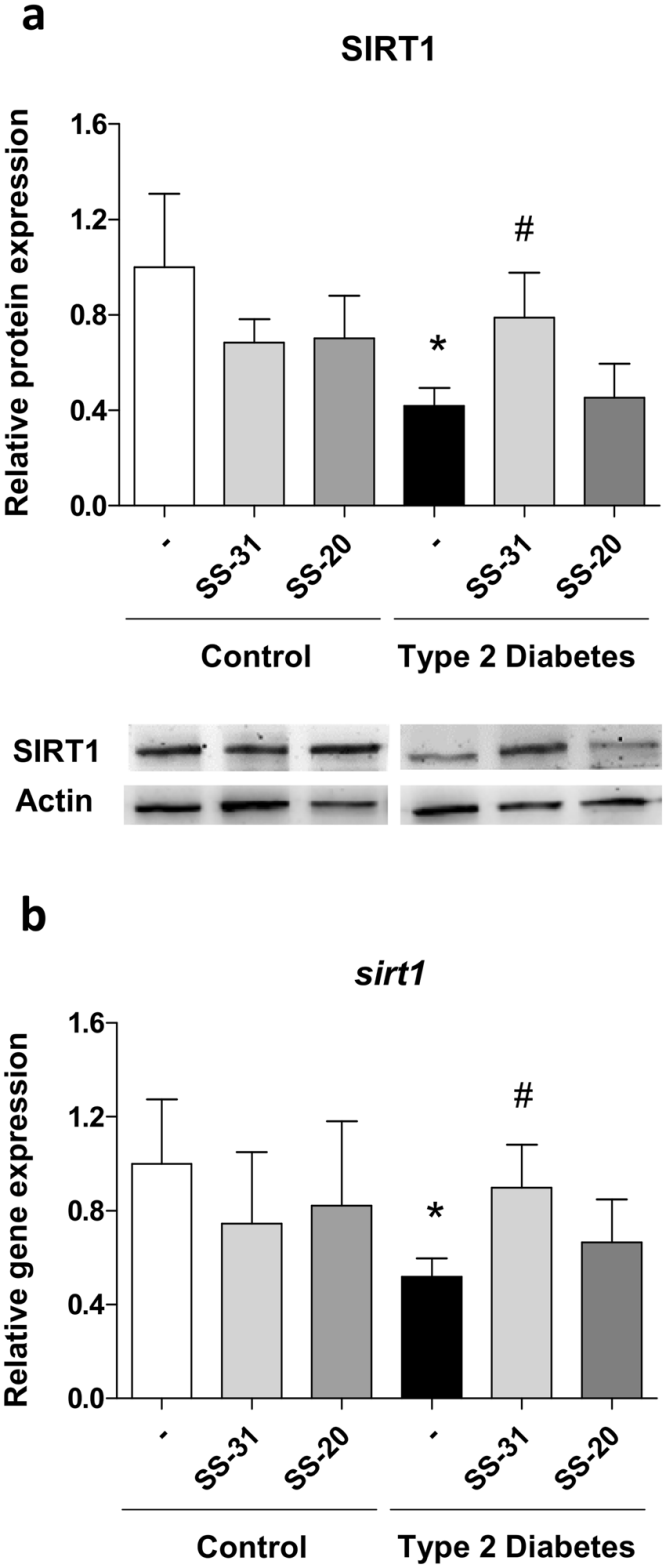
Evaluation of SIRT1 in the absence and presence of SS-31 (30 min, 100 nM) in leukocytes from T2D patients and healthy subjects (**a**) SIRT1 protein levels. (**b**) *sirt1* gene expression. *p < 0.05 with regard to control group ^#^p < 0.05 *vs*. non-treated T2D group.

SIRT1 levels were lower in T2D patients than in controls, both in terms of protein levels (p < 0.05, Fig. 2a) and gene expression (p < 0.05, Fig. 2b). Treatment with SS-31 reversed these effects by enhancing both the protein expression (p < 0.05, Fig. 2a) and gene expression (p < 0.05, Fig. 2b) of SIRT1. SS-20 did not have an effect, highlighting the particular beneficial antioxidant effects of SS-31.

### Leukocyte-endothelium interactions

**Figure 3:**
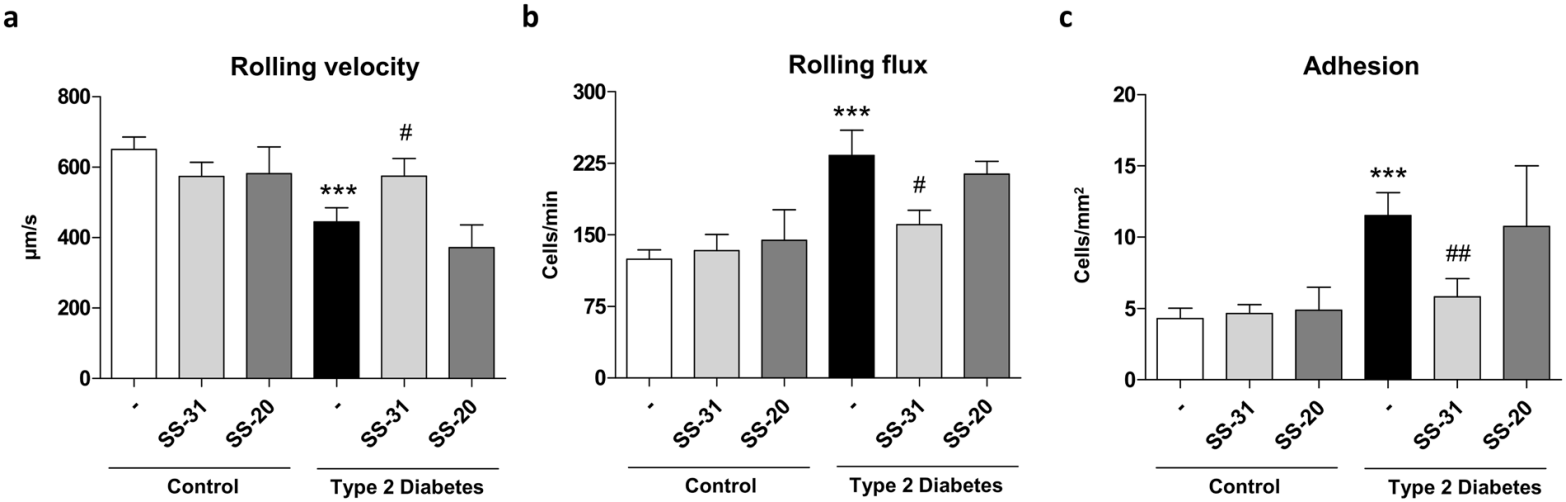
Evaluation of cell interactions in the absence and presence of SS-31 (30 min, 100 nM) in leukocytes from T2D patients and control subjects (**a**) Rolling velocity (μmsecond^−1^) (**b**) Rolling flux (leukocytes per minute) and (**c**) Adhesion (leukocytes per square millimetre). ***p < 0.001 with regard to control group ^#^p < 0.05 ^##^p < 0.01 *vs*. non-treated T2D group.

When we assessed cell interactions in diabetic patients and controls, we observed lower leukocyte rolling velocity (p < 0.001, Fig. 3a) and increased leukocyte adhesion and rolling flux (p < 0.001, Fig. 3b,c) in the latter. Treatment with the mitochondria-targeted antioxidant SS-31 reversed these effects in the leukocytes of T2D patients, triggering a significant increase in leukocyte rolling velocity (p < 0.05, Fig. 3a) and reductions in rolling flux (p < 0.05, Fig. 3b) and adhesion (p < 0.01, Fig. 3c). None of these interactions was influenced by SS-20 treatment (Fig. 3a–c).

### Levels of NFκB-p65 and TNFα

**Figure 4:**
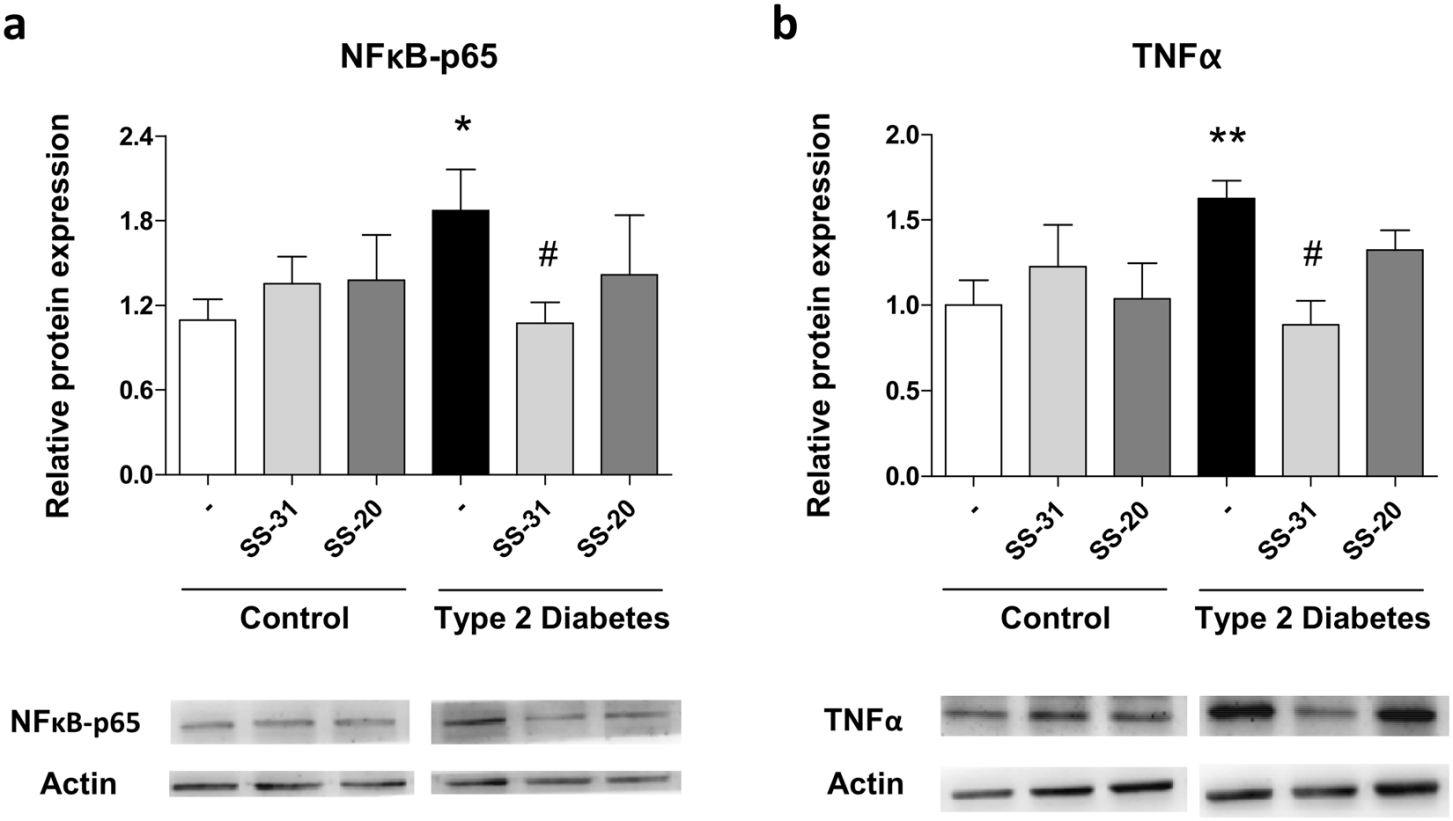
Effects of SS-31 (30 min, 100 nM) on protein expression of NFκB-p65 and TNFα in type 2 diabetic patients and control subjects (**a**) Protein levels of NFκB-p65 and representative WB images (**b**) Protein levels of TNFα and representative WB images. Control samples and T2D samples derived from the same experiment on the one hand and blots on the other were processed in parallel. **p < 0.01 with regard to control group ^#^p < 0.05 *vs*. non-treated T2D group.

Changes in proinflammatory protein expression, measured in terms of NFκB-p65 and TNFα levels, were evaluated in order to determine a possible mechanism by which SS-31 treatment was affecting cell interactions and oxidative stress parameters. A higher peak in NFκB-p65 levels was observed in T2D patients than in healthy subjects (p < 0.05, Fig. 4a). The antioxidant SS-31 decreased inflammation in SS-31-treated leukocytes of T2D patients (p < 0.05, Fig. 4a), while no differences were observed in controls.

TNFα was activated in the diabetic group and not in healthy subjects (p < 0.05, Fig. 4b); in a similar way to NFκB levels, treatment with SS-31 reverted TNFα protein levels to those observed in the control group (p < 0.05, Fig. 4b). Treatment with SS-20 had no effect on NFκB-p65 or TNFα protein levels. Representative western blot images are shown for both results.

## Discussion

In the present study we have observed that a series of parameters that are altered by T2D - oxidative stress, mitochondrial membrane potential and SIRT1, leukocyte-endothelial interactions and NFκB and TNFα levels in leukocytes - are restored by the mitochondrial-targeted antioxidant SS-31, thus highlighting its potential as an agent in the treatment of T2D.

The pathophysiology of T2D involves a series of systemic interrelated alterations including oxidative stress, mitochondrial dysfunction and inflammation. These alterations are a consequence of the metabolic imbalance that occurs in insulin resistance-related diseases and which alter not only glucose levels, but also lipid metabolism^22^. In line with this, our cohort of T2D patients displayed an unfavourable metabolic profile characterized by increased insulin resistance index, hyperglycaemia and atherogenic dyslipidemia manifested by reduced HDL-c and increased triglyceride levels with respect to the control group. Furthermore, chronic inflammation was evident in a rise of hs-CRP levels in our T2D patients.

Oxidative stress is a hallmark of diabetes, and hyperglycaemia is the main cause, since mitochondria generate ROS in response to anaerobic glycolysis, a process that is exacerbated in the diabetic state^23^. A massive body of research exists regarding the pathological consequences of ROS damage for endothelial, cardiac, and blood cells, which precedes the onset of diabetes-related macro- and microvascular complications^17,24^. In the present study we have witnessed how leukocytes from T2D patients display features of oxidative stress, including enhanced total and mitochondrial ROS production and decreased GSH content. Furthermore, the mitochondrial membrane potential of leukocytes from T2D patients was inferior to that of control subjects, thus reflecting impaired mitochondrial function. These findings are in accordance with previous reports by our group^10,25^.

In relation with mitochondrial function, SIRT1 is a key player in the regulation of mitochondrial metabolism^4^ and regulates insulin secretion, adipogenesis and myogenesis. Furthermore, SIRT1 modulates various phases of glucose metabolism in different tissues, including the liver, adipose tissue, pancreas and muscle^5^. In the present study, we have observed a decrease in SIRT1 protein levels and *sirt1* gene expression, suggesting once again an impairment of mitochondrial metabolic function.

Antioxidant therapy for age-related diseases in whose pathophysiology oxidative stress is a crucial player - such as diabetes - has been the subject of extensive research^26,27^. In particular, SS-31, a small cell-permeable small peptide that targets the inner mitochondrial membrane, has been shown to protect different cell types from oxidative stress by scavenging ROS and reducing mitochondrial ROS production^18^. SS-31 concentrates approximately 5,000-fold in mitochondria^28^ and binds specifically to cardiolipin, an essential phospholipid that preserves the structure and proper function of the inner mitochondrial membrane^29^. In the present study, we have observed that treatment of T2D patient leukocytes with SS-31 reduced mitochondrial oxidative stress by decreasing ROS production and increasing GSH levels and membrane potential to similar values to those seen in control leukocytes. In line with this, we have previously reported that mitochondria-targeted antioxidants such as mitoquinone (mitoQ) are of therapeutic value in diabetes because they protect against oxidative damage^30^. Indeed, we have shown that SS-31 increases SIRT1 protein levels and gene expression, suggesting an improvement of mitochondrial function. In fact, experimental evidence of SIRT1 overexpression suggests that a decrease in serum insulin and cholesterol occurs in addition to that of adipose tissue volume and obesity-induced insulin resistance^31^.

The cytoprotective mechanisms of SS-31 are well characterized and include ROS scavenging and inhibition of mitochondrial permeability transition, which maintains mitochondrial ATP synthesis and mitochondrial oxygen consumption^32,33^. In addition, SS-31 inhibits the peroxidase activity of endogenous cyt c, favouring its electron-carrying function^33^.

Different preclinical studies endorse the therapeutic potential of SS-31 in a variety of diseases associated with bioenergetic failure^33^. In relation with diabetes, Tomas *et al*. demonstrated that transplantation of pancreatic islets pre-treated with SS-31 to diabetic mice rapidly restored normoglycaemia (1 day after transplantation), which was sustained at least 14 days after the intervention^18^. Furthermore, it has been reported that SS-31 protects retinal structures during diabetes in a rat model^34^.

In the present study, we have studied in further depth how the antioxidant properties of SS-31 affect the behaviour of leukocytes of T2D patients during the inflammatory state. To do this, we have evaluated leukocyte-endothelial cell interactions in a parallel flow chamber *in vitro* model that mimics leukocyte traffic in the bloodstream. Our results reveal that, whereas T2D induces a decrease in leukocyte rolling velocity and an increase in rolling flux and adhesion, treatment of leukocytes with SS-31 restores these parameters. This highlights how SS-31 ameliorates the enhanced leukocyte-endothelial interactions which precede atherosclerosis in T2D. In line with this, SS-31 has been shown to have a beneficial effect on macrophages by preventing their conversion to foam cells, which are the main mediators of atherogenesis^35^. In addition, it has been reported that SS-31 ameliorates cardiac hypertrophy, diastolic dysfunction and fibrosis in a mice model of hypertensive cardiomyopathy^36^. The benefits of SS-31 for the cardiovascular system may involve several intracellular pathways, such as the inflammatory signalling cascade, which is closely related to ROS production. In this sense, treatment with SS-31 has been shown to prevent IκB degradation and to thus inhibit NFκB activity in TNFα-induced inflammation in myotubes^37^. Moreover, Hao *et al*. demonstrated that SS-31 inhibits ox-LDL-induced inflammation in macrophages, manifested by reduced protein levels of interleukin 6 (IL-6) and TNFα^35^. In accordance with these findings, our data demonstrate that SS-31 treatment decreases NFκB-p65 and TNFα protein levels in the leukocytes of T2D patients, which are initially higher than in those of healthy subjects. The role of ROS as drivers of NFκB activation^38^ is well documented, and antioxidants have been shown to prevent NFκB activation^39,40^. In addition, NFκB itself regulates the expression of a variety of antioxidant genes, highlighting the bidirectional relationship between ROS levels and NFκB activity^41^.

Since NFκB is a major regulator of pro-inflammatory cytokines, we hypothesize that the aforementioned reduction of NFκB-p65 protein levels and, as a consequence, proinflammatory cytokine TNFα expression by SS-31 is responsible, at least in part, for the lower adhesiveness of leukocytes in T2D patients through an inhibition of the cytokine-driven leukocyte-endothelial cell interaction.

We should highlight the possible interaction between anti-diabetic medication and the experimental drug as a potential limitation of the study; diabetic patients are under treatment, and so we cannot rule out a possible synergistic effect of these compounds, which needs to be explored in future research. In summary, our results highlight the potential benefits of the mitochondria- targeted antioxidant SS-31 for the leukocytes of T2D patients; namely, it increases SIRT1 levels and reduces oxidative stress, NFκB pro-inflammatory signalling and proinflamatory cytokine TNFα levels, all of which eventually diminishes leukocyte-endothelial interaction. Our findings suggest that SS-31 can reduce the risk of CVD in T2D patients and should be investigated as a potential new agent to be used in the treatment, not only of diabetes, but of other cardiometabolic disorders.

## Methods

### Subjects

We recruited 51 T2D patients and 57 voluntary controls adjusted for sex and age from the Service of Endocrinology and Nutrition of University Hospital Doctor Peset (Valencia, Spain). All subjects were informed before signing a written consent form. The Ethics Committee of Clinical Investigation of the University Hospital Doctor Peset approved all protocols (ID: 97/16), and the study was conducted in accordance with the ethical principles of the Helsinki Declaration.

Type 2 diabetes was diagnosed following the criteria of the American Diabetes Association. Subjects were excluded from the study when any of the following criteria was met: autoimmune disease; morbid obesity; history of CVD (as peripheral vascular disease, ischemic heart disease, stroke and chronic disease related to cardiovascular risk); infectious, haematological, malignant, organic, or inflammatory disease; or insulin treatment.

Blood samples were collected under fasting conditions from the antecubital vein in a routine blood extraction. Analytical and anthropometric assessments were determined, including weight (kg), height (m), waist circumference (cm), BMI (kg/m^2^), and diastolic and systolic BP (mmHg).

### Biochemical parameters

Venous blood was extracted into Vacutainer® tubes and centrifuged at 1500 g for 10 min at 4 °C, after which biochemical parameters were evaluated as above mentioned^24^. To quantify triglycerides, glucose and total cholesterol levels in serum, was employed an enzymatic method. Insulin levels were measured by immunochemiluminescence and homeostasis model assessment (HOMA-IR = [fasting insulin (μU/ml) x fasting glucose (mg/dl)]/405) was used to calculate insulin resistance. An automatic glycohemoglobin analyser (Arkray Inc., Kyoto, Japan) was used to determine the percentage of HbA1c. A Beckman LX-20 autoanalyzer (Beckman Coulter, La Brea, CA, US) was employed to assesed high density lipoprotein (HDL) levels, low density lipoprotein (LDL) content was estimated with Friedewald’s formula, and high-sensitive C-reactive protein (hs-CRP) levels were measured by an immunonephelometric assay (Behring Nephelometer II, Dade Behring, Inc., Newark, DE, USA).

### Cells

Citrated blood samples were incubated with dextran (3%) for 45 min in order to isolate human polymorphonuclear leukocytes (PMNs). The supernatant was dropped over Ficoll-Hypaque (GE Healthcare, Barcelona, Spain) and centrifuged for 25 min at room temperature at 650 g. Lysis buffer was added to the erythrocytes remaining in the pellet, which was incubated at room temperature for 5 min and then spun at 240 g for 5 min. Leukocytes were washed twice and resuspended at 37 °C in Hanks’ balanced salt solution (HBSS; Sigma Aldrich, MO). Scepter 2.0 cell counter (Millipore, MA, USA) was employed to count cells. Each cellular suspension was divided into two samples, one of which was treated for 30 min with 100 nM of SS-31, and the other with SS-20 in identical conditions (these concentrations did not affect the viability of the cells).

### Measurement of ROS production, membrane potential and glutathione content

Total and mitochondrial ROS production, membrane potential and glutathione (GSH) content were assessed with a fluorescence microscope (IX81; Olympus, Hamburg, Germany) and the analysis was performed with the static cytometry software “ScanR” (Olympus, Hamburg, Germany).

Leukocytes were resuspended (1 × 10^6^ cells/ml) in HBSS and seeded in 48-well plates in duplicate, after which they were maintained for 30 min in a humidified chamber at 5% CO_2_. Cells were then incubated with the respective fluorescence probe. Total ROS production in leukocytes was evaluated with the fluorescence probe 2′,7′-dichlorodihydrofluorescein diacetate (DCFH-DA, 5 × 10^−6^ mol/l, 30 min) and mitochondrial superoxide was detected with MitoSOX fluorochrome (5 × 10^−6^ mol/l, 30 min). GSH content was estimated following incubation with the fluorochrome 5-Chloromethylfluorescein Diacetate (CMFDA, 2.5 × 10^−6^ mol/l, 30 min). The fluorescent dye tetramethylrhodamine methyl ester (TMRM, 5 × 10^−6^ mol/l, 30 min) was used to assess mitochondrial membrane potential. A total of 12 images per well were recorded, nuclei were visualized with Hoechst 33342 (Sigma Aldrich, MO), and results were expressed as arbitrary fluorescence units.

### Confocal Microscopy

Isolated leukocytes were resuspended (1 × 10^6^ cells/ml) in HBSS and plated in slide (chambered coverslip) with 8 wells and maintained for 30 min in a humidified chamber at 5% CO_2_. Then, cells were loaded with the respective fluorochrome for 30 min (MitoSOX, (5 × 10^−6^ mol/l); TMRM, (5 × 10^−6^ mol/l); Nonyl acridine orange, NAO (1 × 10^−6^ to stain mitochondria)) in HBSS, 37 °C. Thereafter, staining solution was replaced with fresh HBSS and cells were visualized. The excitation wavelengths for flurochromes were 510 for MitoSOX, 548 for TMRM, and 485 for NAO. Emission filters was adjusted to 556–600 nm for MitoSOX, 565–603 nm for TMRM, and 505–540 nm for NAO.

Confocal images were acquired using a Leica TCS-SP8 confocal laser scanning unit (*Leica* Microsystems, Mannheim, Germany). The white light laser (WLL) source of the Leica TCS-SP8 perfectly matches the spectral properties of any fluorophore excitable in the visible spectral range. The tunning range covers 470 to 670 nm. Imaging was recorded with a 63x/1.4 oil immersion confocal objective and images for each fluorophore were recorded sequentially to avoid channel spill-over.

A complex I inhibitor (rotenone, 50 μM) was used as a positive control for mitochondrial superoxide production (MitoSOX), and a mitochondrial uncoupling agent (carbonyl cyanide m-chlorophenyl hydrazone, CCCP, 25 μM) was used as a positive control for mitochondrial depolarization (TMRM).

### Leukocyte-endothelium interaction assays

Adhesion assays were performed using an *in vitro* model with parallel plate flow chamber. Once a human umbilical vein endothelial cells (HUVEC) confluent monolayer had been harvested from fresh umbilical cords by means of collagenase (1 mg/ml in phosphate-buffered saline; Thermo Fisher Scientific, MA) digestion for 17 min, HUVEC primary cultures were grown over fibronectin-coated plastic coverslips (Sigma Aldrich, MO) and incubated with complete EMB-2 culture medium (Lonza, Basel, Switzerland). Next, we placed these coverslips into the bottom of the flow chamber, so that a portion (5 × 25 mm) of the HUVEC monolayer was exposed, and the flow chamber was settup on an inverted microscope (Nikon Eclipse TE 2000-S; Amstleveen, The Netherlands) connected to a video camera (Sony Exware HAD; Koeln, Germany). Leukocyte suspensions were perfused across the endothelial monolayer at a flow rate of 0.36 ml/min. Real time images of the flow-exposed monolayer were recorded for 5 min and analyzed. Leukocyte rolling flux, rolling velocity and adhesion were determined as described elsewhere^25^.

### Western Blot analysis

Leukocytes were incubated for 15 min on ice with a lysis buffer (400 mM NaCl, 20 mM HEPES pH 7.5, 0.1 mM EDTA, 20% glycerol, 10 μM Na_2_MoO_4_ and 0.5% Nonidet P-40) containing protease inhibitors (10 mM β-glycerolphosphate, 10 mM NaF, 10 mM PNP and 1 mM Na_3_VO_4_) and dithiothreitol 1 mM and centrifuged at 4 °C for 15 min. Protein concentrations were determined using the BCA protein assay kit (Thermo Fisher Scientific, IL, US). Protein samples (25 µg) were resolved by means of sodium dodecyl sulfate polyacrylamide gel electrophoresis and then transferred to nitrocellulose membranes. After blocking, they were incubated with primary antibodies overnight at 4 °C. We used primary antibodies: anti-NFκB-p65 (phospho S536) rabbit polyclonal antibody (Abcam, Cambridge, MA), anti-TNFα rabbit polyclonal antibody, anti-SIRT1 rabbit polyclonal antibody (Millipore Iberica, Madrid, Spain) and anti-actin rabbit polyclonal antibody (Sigma Aldrich, Missouri, US). Blots were incubated with the secondary antibody HRP goat anti-rabbit (Millipore Iberica, Madrid, Spain) and were developed for 2 min with supersignal west femto (Thermo Fisher Scientific, IL, US) or ECL plus reagent (GE Healthcare, LC, UK). Chemiluminescence signals were detected with a Fusion FX5 acquisition system (Vilbert Lourmat, Marne La Vallée, France) and analysed by densitometry using Bio1D software (Vilbert Lourmat, Marne La Vallée, France), and protein bands were normalized to the expression of actin in the same sample.

### Quantitative RT-PCR (qRT-PCR)

Total RNA was extracted from leukocyte pellets using the GeneAll® RibospinTM kit (GeneAll Biotechnology, Hilden, Germany). RNA concentrations were determined using Nanodrop 2000c (Thermo Fisher Scientific, Waltham, MA), and an optical density absorption ratio of 260/280 nm between 1.8 and 2.0 confirmed RNA purity. A reverse transcriptase-polymerase chain reaction assay was performed with 1 μg of mRNA employing a RevertAid First Strand c-DNA Synthesis kit (Thermo Fisher Scientific, Waltham, MA). In the following steps, 1 μl of first-strand cDNA was used. *Sirt1* and *gapdh* (housekeeping internal control) gene expression were measured by qRT-PCR using the KAPA SYBR FAST universal master mix (Biosystems, MA) in a 7500 Fast real-time PCR system (Life Technologies, California, US). Methodological procedure details and primer sequences are shown in Table 2. Relative quantification analysis was achieved using the comparative 2^−ΔΔCt^ method with Expression Suite software (Life Technologies, California, US).

**Table 2:** Protocol details and primers sequences.

qRT-PCR protocol				
Temperature	95 °C	95 °C	60 °C	Melting
Time	10 min	10 s	30 s	curve
N^o^. of Cycles	1	40		
**Primers**				
**Target**	**Direction**	**Sequence (5′-3′)**		
*Sirt1*	Forward	TGGGTACCGAGATAACCTTCT TGTTCGAGGATCTGTGCCAA		
	Reverse			
*Gapdh*	Forward	CGCATCTTCTTTTGCGTCG TTGAGGTCAATGAAGGGGTCA		
	Reverse			

### Statistical analyses

Data analysis was carried out with SPSS 17.0. In the table, the results for parametric variables are expressed as mean ± SD and median (25^th^ and 75^th^ percentiles) for non-parametric variables. Bar graphs in figures indicate mean ± SEM. A The Mann–Whitney U test was used for comparisons between 2 groups in non-normally distributed samples, the student’s t test was used for normally distributed samples, and the Chi-Square test was used for proportions. Changes in biochemical parameters were analyzed using BMI as a covariate in a univariate general linear model. When evaluating the effects of SS-31 and SS-20, data were compared with a one-way analysis of variance (ANOVA) followed by Student–Newman–Keuls post hoc test. We have considered significant differences when p < 0.05.

## Electronic supplementary material

Supplementary File
