# Supplementary material for: The mitochondrial antioxidant SS-31 increases SIRT1 levels and ameliorates inflammation, oxidative stress and leukocyte-endothelium interactions in type 2 diabetes
Source: Sci Rep. 2018 Oct 26;8:15862. doi: 10.1038/s41598-018-34251-8 (PMC6203778; doi:10.1038/s41598-018-34251-8)

The mitochondrial antioxidant SS-31 increases SIRT1 levels and ameliorates inflammation, oxidative stress and leukocyte-endothelium interactions in type 2 diabetes

Running title: SS-31 as a novel potential therapy for type 2 diabetes

Irene Escribano-Lopez[1], Noelia Diaz-Morales[1], Francesca Iannantuoni[1], Sandra Lopez-Domenech[1], Aranzazu M de Marañón[1], Zaida Abad-Jimenez[1], Celia Bañuls[1], Susana Rovira-Llopis[1], Jose R Herance[2], Milagros Rocha[1,3*], Victor M Victor[1,3,6*]

Supplementary Figure S1

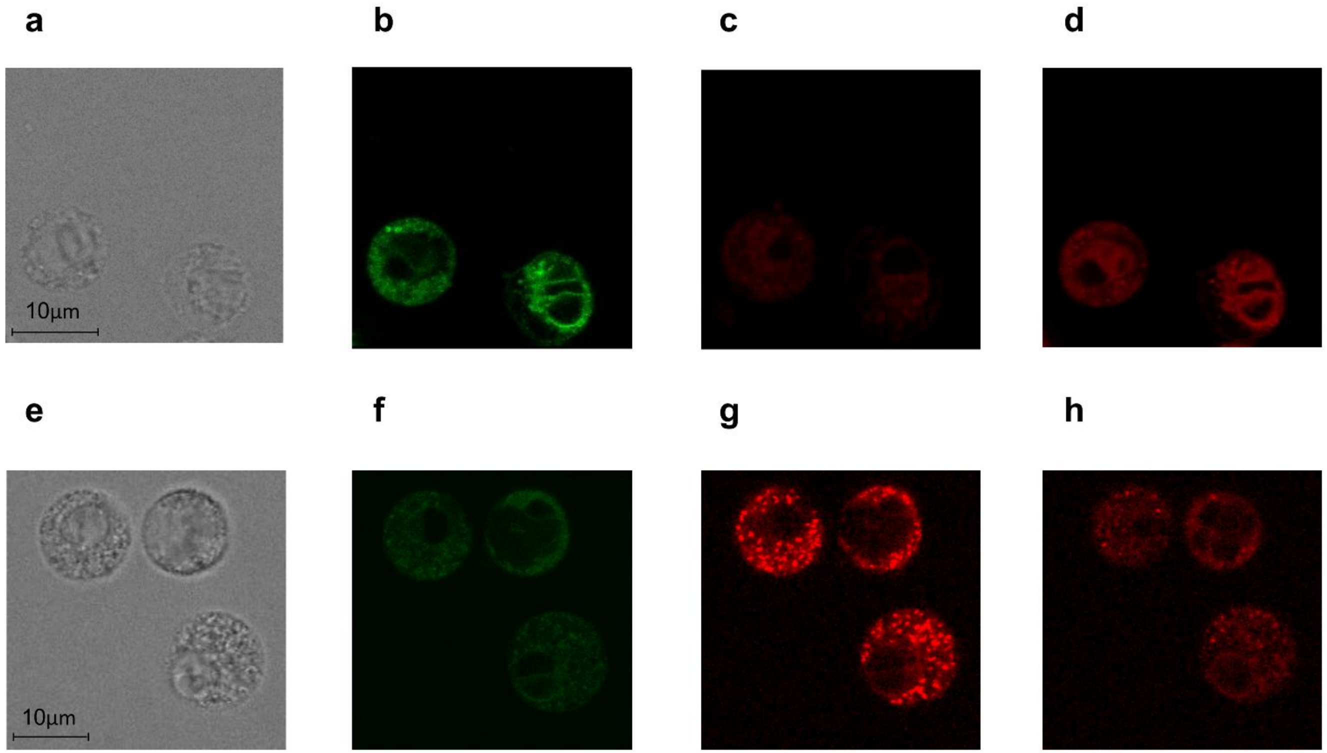

Supplementary Figure S1. Representative confocal microscopy images in human leukocytes (a) Images taken by light microscopy (transmission) **(b)** Mitochondrial staining with cardiolipin-sensitive fluorescent dye NAO **(c)** Mitochondrial superoxide assessed as MitoSOX fluorescence **(d)** Stimulated mitoSOX fluorescence after a complex I inhibitor addition (rotenone, 50µM) **(e)** Images taken by light microscopy (transmission) **(f)** Mitochondrial staining with cardiolipin-sensitive fluorescent dye NAO **(g)** Mitochondrial membrane potential measured as TMRM fluorescence **(h)** TMRM fluorescence after mitochondrial uncoupling agent supply (CCCP, 25µM)

Supplementary Information

NFκB-p65 (ORIGINAL BLOTS)

- Representative WB images for controls

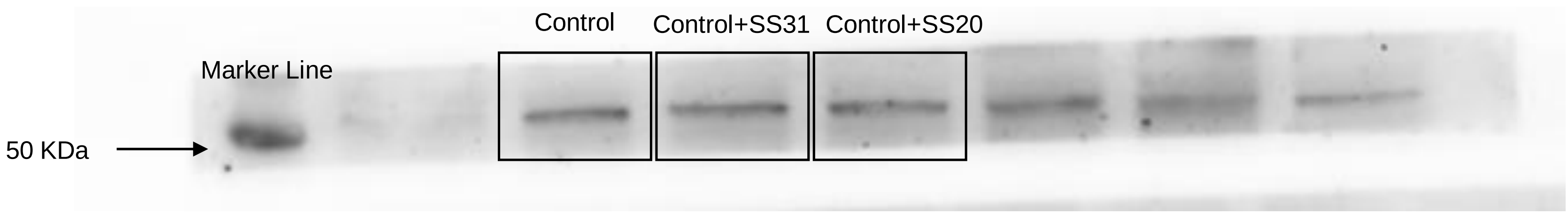

- Representative WB images for T2D patients

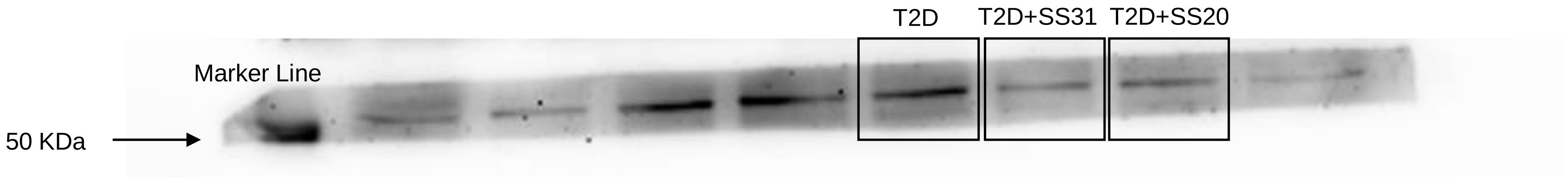

ACTIN - NFκB-p65 (ORIGINAL BLOTS)

- Representative WB images for controls

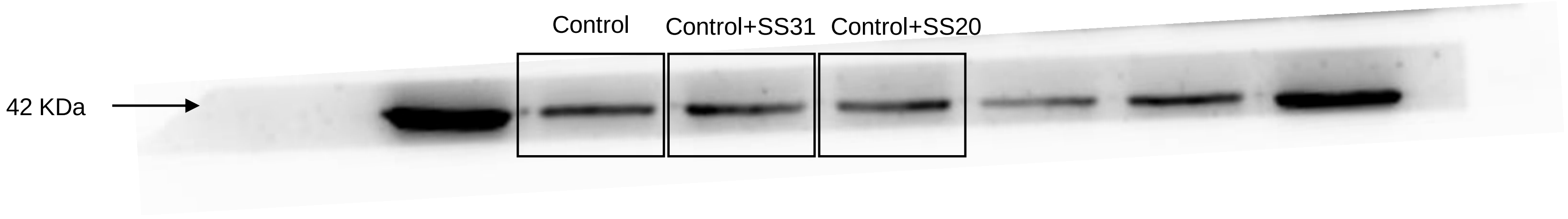

- Representative WB images for T2D patients

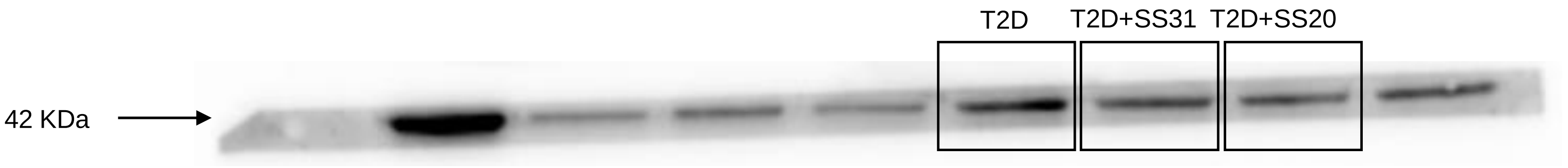

TNFα (ORIGINAL BLOTS)

- Representative WB images for controls

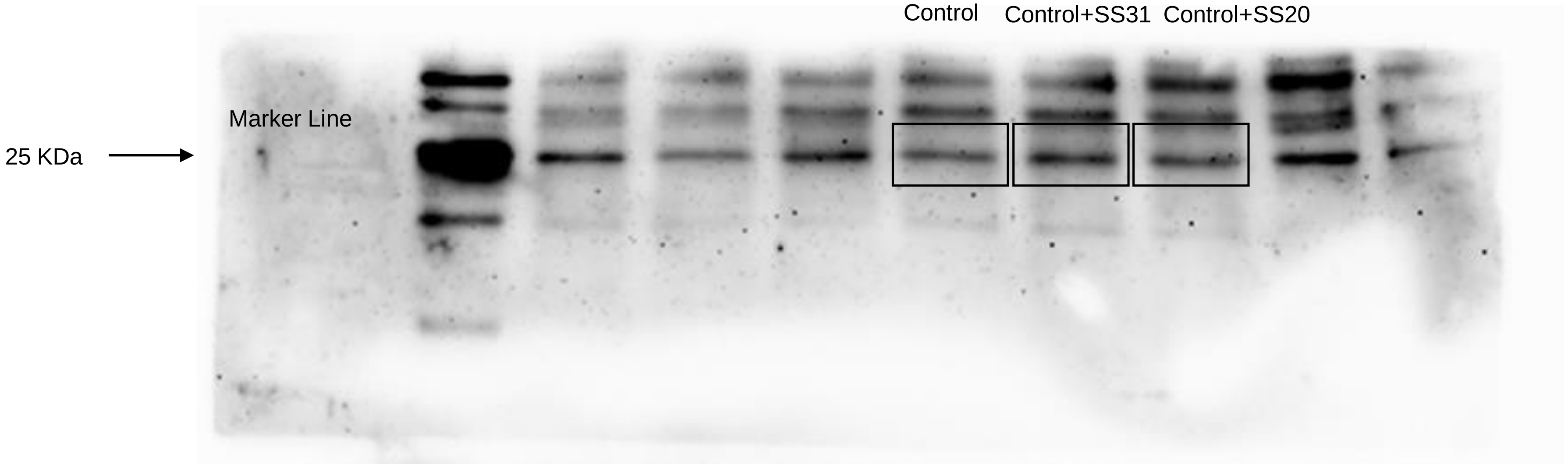

- Representative WB images for T2D patients

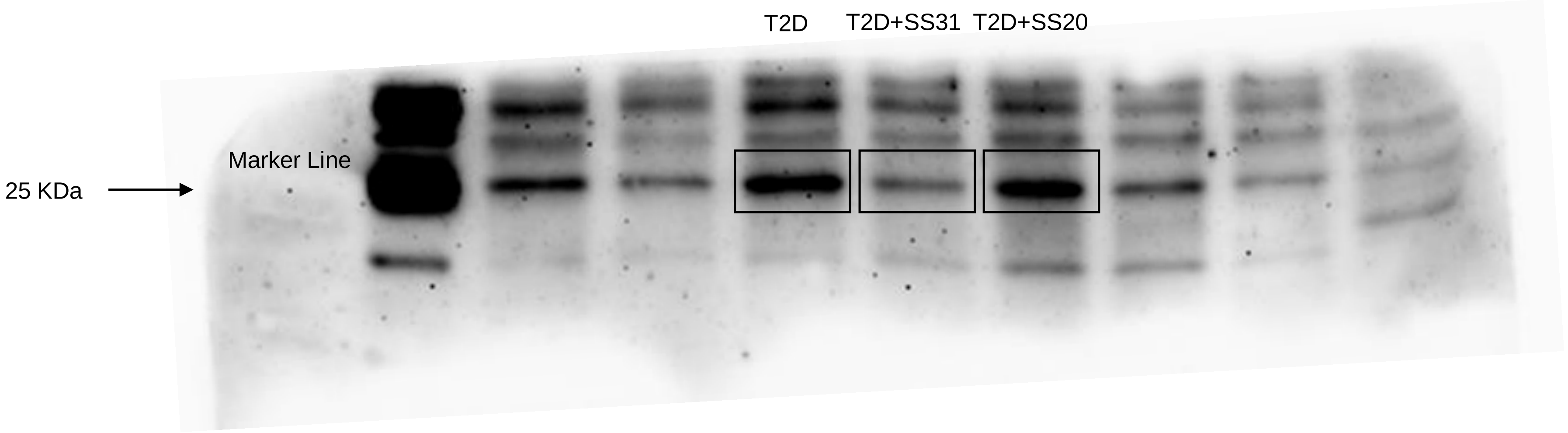

ACTIN - TNFα (ORIGINAL BLOTS)

- Representative WB images for controls

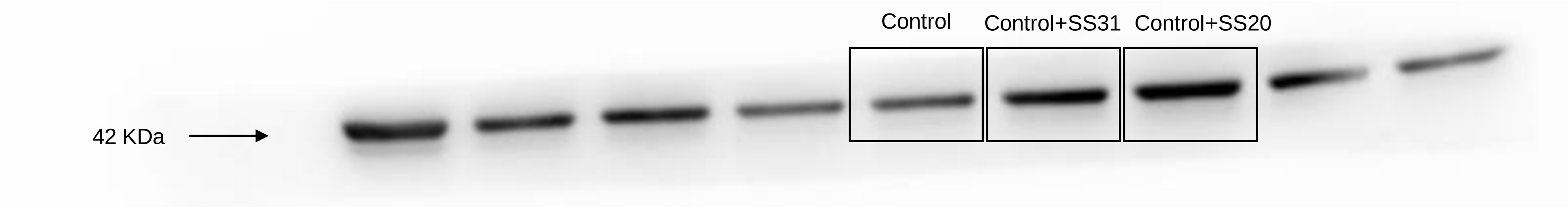

- Representative WB images for T2D patients

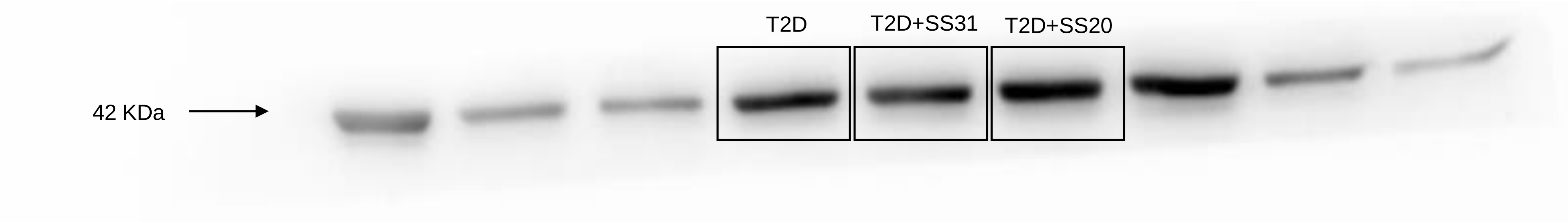

SIRT1 (ORIGINAL BLOTS)

- Representative WB images for

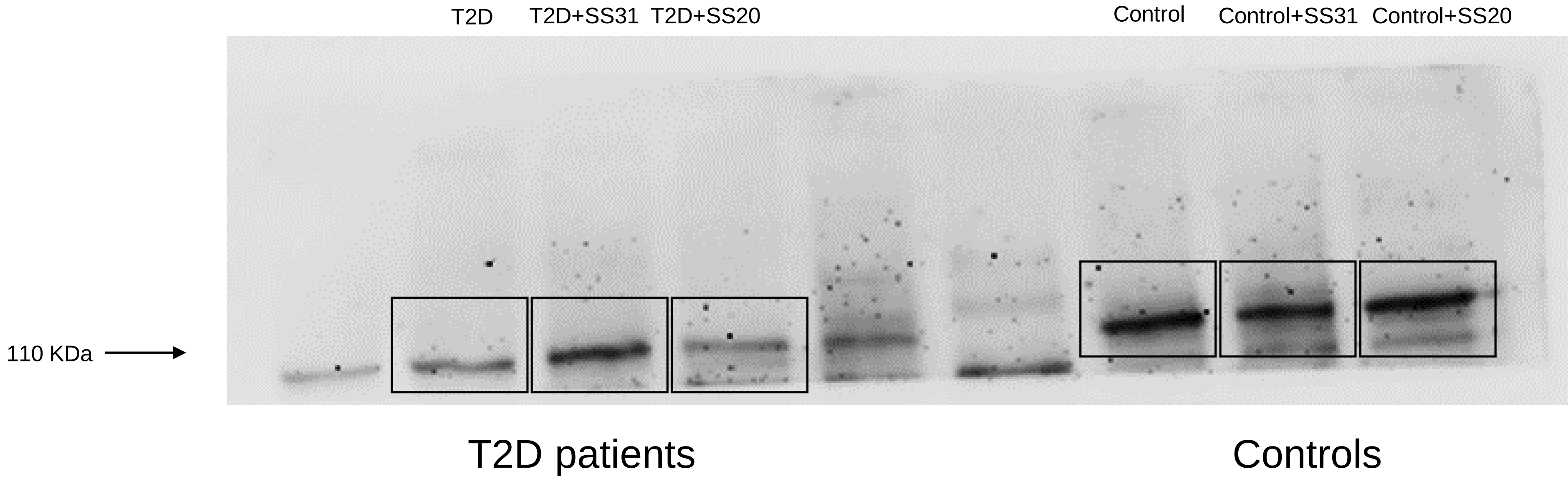

ACTIN– SIRT1 (ORIGINAL BLOTS)

- Representative WB images for

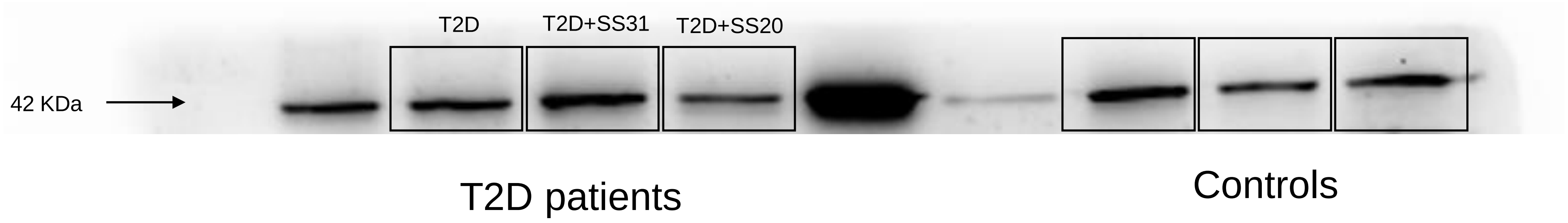

Supplement: Supplementary file 1 — Supplementary File [file 41598_2018_34251_MOESM1_ESM.pdf]
